# Therapeutic Effectiveness of Anti-RAGE Antibody Administration in a Rat Model of Crush Injury

**DOI:** 10.1038/s41598-017-12065-4

**Published:** 2017-09-25

**Authors:** Hisatake Matsumoto, Naoya Matsumoto, Junya Shimazaki, Junichiro Nakagawa, Yukio Imamura, Kazuma Yamakawa, Tomoki Yamada, Mitsunori Ikeda, Hiroko Hiraike, Hiroshi Ogura, Takeshi Shimazu

**Affiliations:** 1 Department of Traumatology and Acute Critical Medicine, Osaka University Graduate School of Medicine, 2-15 Yamadaoka Suita, Osaka, 565-0871 Japan; 2 Laboratory of Nano-Bio Probes, RIKEN Quantitative Biology Center, 6-2-3 Furuedai, Suita, Osaka, 565-0874 Japan

## Abstract

Crush injury patients often have systemic inflammatory response syndrome that leads to multiple organ failure. Receptor for advanced glycation endproducts (RAGE) functions as a pattern recognition receptor that regulates inflammation. We evaluated the effects of anti-RAGE antibody in a crush injury model. Pressure was applied to both hindlimbs of rats for 6 h by 3.0-kg blocks and then released. Animals were randomly divided into the sham (RAGE-Sh) group, crush (RAGE-Ctrl) group or anti-RAGE antibody-treated crush (RAGE-Tx) group. Samples were collected at 3, 6 and 24 h after releasing pressure. In the RAGE-Ctrl group, fluorescent immunostaining in the lung showed upregulated RAGE expression at 3 h. The serum soluble RAGE (sRAGE) level, which reflects the amount of RAGE expression in systemic tissue, increased at 6 h. Serum interleukin 6 (IL-6; systemic inflammation marker) increased immediately at 3 h. Histological analysis revealed lung injury at 6 and 24 h. Administration of anti-RAGE antibody before releasing compression inhibited upregulated RAGE expression in the lung alveoli, suppressed RAGE-associated mediators sRAGE and IL6, attenuated the lung damage and improved the 7-day survival rate. Collectively, our results indicated that the use of anti-RAGE antibody before releasing compression is associated with a favourable prognosis following crush injury.

## Introduction

Crush injury is generally observed in large earthquakes, in wars and acts of terrorism and in industrial and traffic accidents^1,2^. Clinical manifestations in the acute phase after crush injury are mainly hypovolemia, lethal arrhythmias and acute renal failure^3,4^. Even though adequate fluid volume restoration and renal replacement therapy are conducted, crush injury patients often have systemic inflammatory response syndrome (SIRS) and progress to multiple organ failure (MOF), which leads to death^5^. Mortality in crush syndrome patients is high, around 13–14% in previous reports^3,6,7^, and thus, crush syndrome is a life-threatening condition that must be addressed. However, the pathophysiological mechanism of and effective therapies for SIRS that lead to MOF after crush injury have not been elucidated. Previously, we developed a rat model of crush injury and assessed the pathogenesis of acute inflammation after crush injury^8,9^. Recently, we reported that high-mobility group box 1 (HMGB-1), which is one of the damage-associated molecular patterns (DAMPs), increases in reaction to tissue damage related to crush injury and plays a role as a proinflammatory mediator and that anti-HMGB-1 antibody inhibits systemic inflammation and improves survival^10^. HMGB1 has been reported to promote signalling of the receptor for advanced glycation endproducts (RAGE). Therefore, we hypothesised that RAGE signalling might play an important role in the pathogenesis of acute inflammation after crush injury.

RAGE is known to gradually accumulate in diabetes and is thought to be one of the pattern recognition receptors^11^. RAGE signalling promotes inflammatory mediators and has been reported to be actively involved in the progression of chronic inflammatory and age-related diseases such as arteriosclerosis and Alzheimer’s disease^12–14^. It is now emerging that RAGE signalling is also related to the pathogenesis of acute inflammatory diseases^11,15^. RAGE has been shown to be involved in both innate and adaptive immune response systems and is expressed in a wide range of cell types such as systemic endothelial cells and various types of leukocytes^16,17^. Interaction between RAGE and its ligands such as HMGB-1 has been implicated in the activation of multiple signalling pathways for immune responses and the subsequent development of SIRS associated with sepsis and acute lung damage^11,18,19^.

RAGE exists in two forms, as a trans-membrane signalling receptor and in a soluble form called sRAGE. Mounting evidence indicates that the amount of sRAGE reflects that of systemic tissue RAGE expression and the inflammatory process induced by RAGE signalling^19–22^. sRAGE is composed of two forms. One isoform is called the endogenous secreted form of RAGE (esRAGE). This isoform is identified as a splicing variant, which is secreted directly into the circulation without anchoring to the plasma membrane^23^. The other form, cleaved RAGE (cRAGE), is generated through the cleavage of cell membrane-anchored RAGE by proteolytic enzymes such as matrix metallopeptidase 9 (MMP-9)^24^. Previously, we reported that the serum level of sRAGE increased in early sepsis, thus reflecting the severity of the sepsis, and showed that sRAGE was mainly derived from cRAGE^25^. Several reports that have shed light on the functional role of RAGE in acute inflammatory diseases are consistent with our results, suggesting that cRAGE is induced in an early systemic stress response^21,26^.

The purposes of this study were to investigate RAGE signalling in systemic acute inflammation after crush injury and to assess the therapeutic effects of anti-RAGE antibody in a rat model of crush injury.

## Materials and Methods

### Animals

Male Wistar rats free of specific pathogens (260–300 g) were obtained from Nihon SLC, Inc. (Hamamatsu, Japan). All rats had free access to both food and water. All of the experiments were conducted in accordance with the guidelines of the Institutional Animal Care and Use Committee of Osaka University Graduate School of Medicine and were approved by the animal care committee (Permit Number: 26-001-005). Rats were anaesthetised with the intraperitoneal administration of a mixture of midazolam (4 mg/kg), medetomidine (0.3 mg/kg) and butorphanol (5 mg/kg). The animals were fixed in the supine position on a heating pad and were maintained at 37 °C during the experiment as measured by an intra-rectal thermistor probe (Bio-Medica Ltd., Osaka, Japan). A 0.3-mm inner diameter polyethylene tube (Imamura Co., Ltd., Tokyo, Japan) was inserted into the left external jugular vein for fluid replacement and the administration of drugs.

### Crush injury models

**Figure 1:**
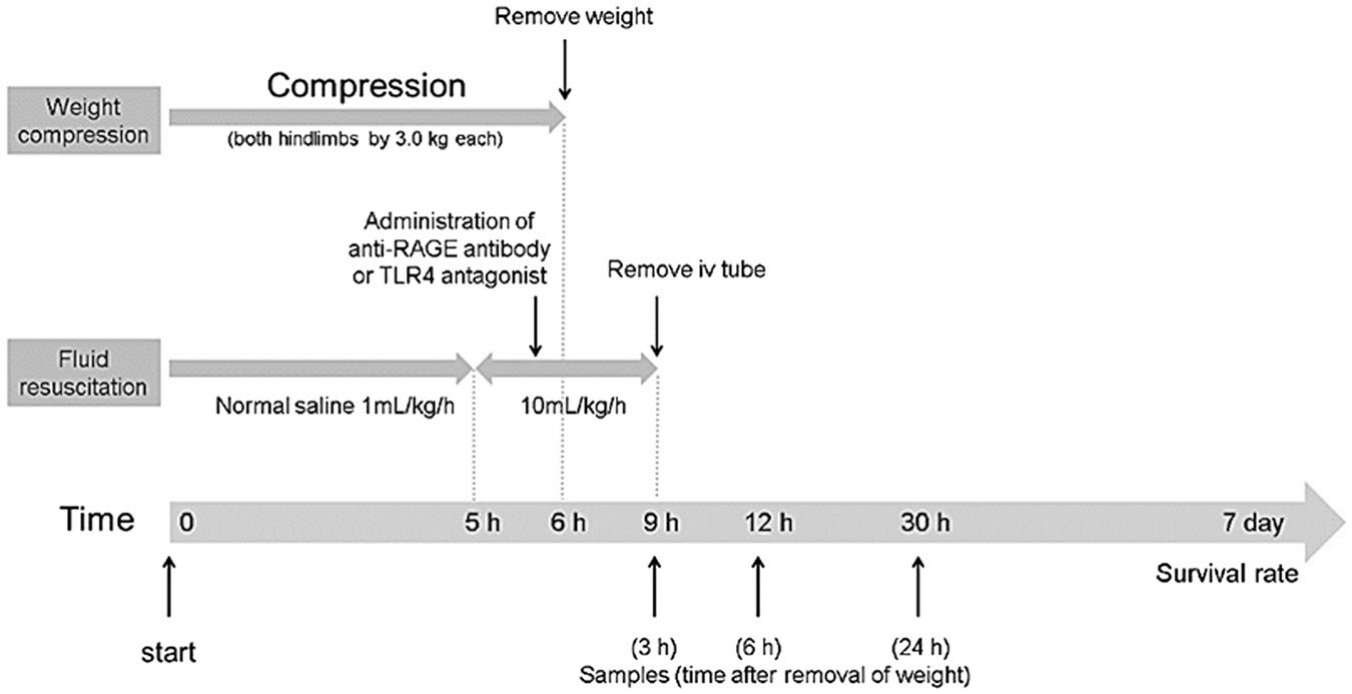
Experimental protocol of the crush injury model in rats.

Crush injury was induced by the method of Akimau *et al*. but with some modification as described previously^8–10,27^ and is shown in Fig. 1. In brief, the bilateral hindlimbs of rats were compressed with a specific apparatus created for the experiment (Asai Works Co., Osaka, Japan; Konan Medical Laboratory Co., Kobe, Japan). The lower part of the apparatus was composed of a rectangular metallic platform (12 × 9 × 1 cm), with four metallic rods (0.9 cm in diameter and 8 cm in height) welded perpendicularly onto the 4 corners. The upper part of the apparatus was composed of a rectangular plastic plate (12 × 9 × 1 cm), and a plastic block (12 × 9 × 1 cm) glued to the plate surface, along its external edge. Four holes of 1 cm in diameter were drilled in the corners of the upper part to match the rods from the platform. Round weights (2.5 kg) were placed on the upper part via a removable metallic rod, which was inserted into a centre hole in the upper part. The total weight of the upper component was 3.0 kg. The bilateral hindlimbs were stretched externally and placed on the platform. The upper components were placed on each platform. To hold the hindlimbs, toes were fixed using tape. The apparatus was removed 6 h after compression, and reperfusion of the hindlimbs was allowed. Continuous intravenous infusion of normal saline via a polyethylene tube (1 mL/kg/h) was administrated during the first 5 h from the start of compression (i.e. up to 1 h before releasing compression) and then at 10 mL/kg/h during the next 4 h (i.e. 1 h before releasing compression until 3 h after releasing compression). After the procedure, the left jugular vein was ligated after removal of the polyethylene tube and the operative wound was closed. The animals were returned to their home cages and given free access to feed and water.

## Experimental design

### Experiment 1

Animals were divided into three groups: sham-operated group (RAGE-Sh group), crush group (RAGE-Ctrl group), or anti-RAGE antibody-treated crush group (RAGE-Tx group). The Sh group followed the above procedure but without compression the hindlimbs. In the RAGE-Tx group, anti-RAGE antibody (rabbit polyclonal IgG; Santa Cruz Biotechnology, Santa Cruz, CA, USA), which is specifically raised against the full 300 amino acid-long protein of RAGE and for which a neutralization effect was shown in a previous study^18^, dissolved in phosphate-buffered saline (PBS; 10 mg/ml) was administered immediately before the removal of the weights at the dose of 350 µg/kg. In the RAGE-Ctrl group, rabbit polyclonal IgG (Santa Cruz Biotechnology) dissolved in the same amount of PBS was injected instead of the anti-RAGE-antibody. Survival rates were evaluated until day 7 following crush injury (n = 6 in the RAGE-Sh group and n = 20 each in both the RAGE-Ctrl and RAGE-Tx groups). Independently, the blood and tissue samples from separate animals were used for evaluation of blood and for histological analysis. Blood samples were taken at 3, 6 and 24 h after release of the compression (n = 4 for each time point in the RAGE-Sh group and n = 7 for each time point in both the RAGE-Ctrl and RAGE-Tx groups). Perfusion fixation with 4% paraformaldehyde was performed at 3, 6 and 24 h after releasing compression (n = 3 for each time point in the RAGE-Sh group and n = 4 for each time point in both the RAGE-Ctrl and RAGE-Tx groups).

### Analyses of related biological parameters in blood

The abdominal cavity was opened under anaesthesia, the retroperitoneum was bluntly exfoliated, and then the blood sample was obtained through an Intramedic PE-50 polyethylene tubing (Becton, Dickinson, Franklin Lakes, NJ, USA) cannulating the abdominal aorta. The serum was isolated by centrifugation at 3000 x *g* for 15 min and stored at −30 °C until use. Enzyme-linked immunosorbent assay (ELISA) kits were used to measure serum concentrations of interleukin 6 (IL-6) (R&D Systems, Minneapolis, MN, USA), HMGB1 (Shino-test, Kanagawa, Japan), sRAGE (Abcam, Cambridge, UK), the endogenous secreted form of RAGE (esRAGE) and soluble vascular adhesion molecule-1 (sVCAM-1) (MyBiosource, San Diego, CA, USA) and MMP-9 (R&D Systems). The frozen samples were thawed and then processed according to the manufacturers’ instructions. Absorbance was measured with a microplate reader (SH-9000Lab; Corona Electric Co., Ltd., Ibaraki, Japan).

### Histological analysis

#### Tissue fixation

All rats were deeply anaesthetised and then perfused transcardially with PBS, followed by cold 4% paraformaldehyde in 0.1 M phosphate buffer (PB). The lung, kidney, liver and brain, which are deeply associated with the development of MOF, were dissected, immersed in the same fixative at 4 °C for 6 hours, and cryoprotected in an increasing concentration of sucrose solutions (15%, 20% and 25% sucrose in 0.1 M PB) at 4 °C for 3 days. After the tissues were frozen in OCT compound (Tissue-Tek; Sakura Finetechnical Co., Ltd., Tokyo, Japan), they were sliced into 8-µm-thick sections by cryostat (CM3050S, Leica Microsystems, Wetzlar, Germany) and mounted onto glass slides for histological observations. Tissue sections were stained with haematoxylin-eosin stain using standard techniques.

#### Fluorescent immunostaining

The sections prepared as mentioned above were incubated with the primary antibody at 4 °C overnight. Incubation in secondary antibody fluorochrome conjugate was performed for 1 h at room temperature. The primary and secondary antibodies were diluted with an antibody dilution buffer (0.5 M NaCl, 3% BSA, 5% normal goat serum, 0.3% Triton X-100, 0.05% NaN_3_ and 0.01 M PB, pH 7.2). After each step, the sections were thoroughly washed in PBS. Finally, they were mounted with 4′, 6-diamidino-2-phenylindole (DAPI) staining solution (Vector Laboratories, Inc., Burlingame, CA, USA) for nuclei staining, coverslipped and incubated at 4 °C for 30 min in the dark.

The primary antibodies were as follows: rat anti-rat RAGE monoclonal antibody (R&D Systems), mouse anti-rat p180 lamellar body protein antibody (Abcam), mouse anti-rat podoplanin (AngioBio, San Diego, CA, USA), mouse anti-rat endothelial cell antibody (anti-RECA1) (Abcam) and mouse anti-rat CD68 antibody (Abcam).

The secondary antibodies used were Alexa Fluor 488 goat anti-mouse IgG and Alexa Fluor 555 goat anti-rat IgG. Negative controls were generated by omitting the primary antibody. In addition, rat IgG2A (R&D Systems) was used as an isotype control for the rat anti-rat RAGE monoclonal antibody. The fluorescence was analysed with a fluorescent microscope (BZ-9000; Keyence Co. Ltd., Osaka, Japan) and a fluorescence microscope with pulse-structured illumination (BZ-700; Keyence Co., Ltd.) to examine the co-localisation of RAGE and other markers. Quantification of the relative intensity was calculated with the use of a BZ-9000 analyser (Keyence Co., Ltd.). Ten fields of alveolar sections were randomly selected in two RAGE-stained slides of each rat, and the relative intensity was examined using a defined rectangular field area (0.38 mm^2^) at a magnification of ×200. The relative intensity of RAGE was calculated as follows: average RAGE intensity = sum of RAGE intensity in each alveolar section/10 fields of the alveolar section. RAGE relative intensity (%) = average RAGE intensity/the defined maximum RAGE intensity.

### Experiment 2

To evaluate the effect of the TLR4 antagonist in the rat model of crush injury, animals were randomly assigned to two groups: crush group (TLR4-Ctrl group) or TLR4 antagonist-treated crush group (TLR4-Tx group). In the TLR4-Tx group, TLR4 antagonist (Chemshene, Monmouth Junction, NJ, USA) was dissolved in dimethyl sulfoxide (DMSO) solvent (Sigma-Aldrich), mixed with PBS (10 mg/ml) and then administered immediately before the removal of the weights at the dose of 2 mg/kg based on previous reports^28,29^. In the RAGE-Ctrl group, DMSO mixed with the same amount of PBS was injected instead of TLR4 antagonist. Blood samples were collected at 6 h after releasing compression (n = 6 in both the TLR4-Ctrl and TLR4-Tx groups), and IL-6 was measured with the ELISA kits. Haematoxylin-eosin staining in the lung was performed after perfusion fixation at 6 h after releasing compression (n = 3 in both the TLR4-Ctrl and TLR4-Tx groups).

### Statistical analysis

Kaplan-Meier survival curves were calculated and compared using log-rank statistics with the Bonferroni correction for multiple comparisons. The results obtained by ELISA are expressed as median and interquartile range, and the results of RAGE relative intensity are expressed as percentages. Statistical analyses were performed with analysis of variance followed by Dunnett multiple comparison test or Student *t*-test. A value of *P* < 0.05 was considered to indicate statistical significance. Statistical analyses were performed with IBM SPSS Statistics version 22.0 for Windows (SPSS Inc., Chicago, IL, USA).

## Results

### Experiment 1

#### Survival

**Figure 2:**
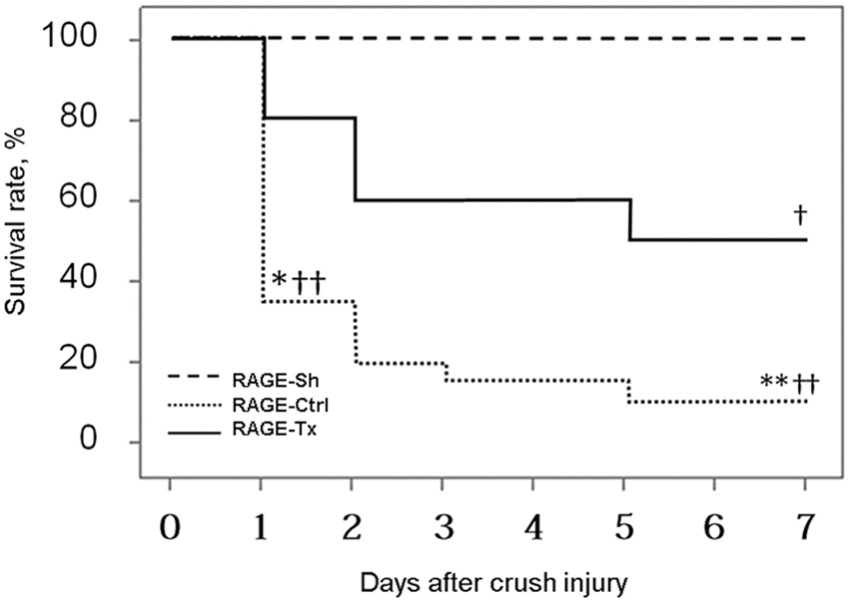
The 7-day survival rates following crush injury. Survival rates at 7 days were 100% in the RAGE-Sh group, 10% in the RAGE-Ctrl group and 50% in the RAGE-Tx group. The survival rate was significantly improved in the RAGE-Tx group versus that in the RAGE-Ctrl group. RAGE-Sh group (n = 6), RAGE-Ctrl group (n = 20), RAGE-Tx group (n = 20). *P < 0.05, **P < 0.01 vs. the RAGE-Tx group. ^†^ *P* < 0.05, ^††^ *P* < 0.01 vs. the RAGE-Sh group.

Kaplan-Meier survival curves are shown in Fig. 2. In the RAGE-Sh group, all rats survived throughout the experiment. The survival rate within the first 24 h after crush injury was 35% (7/20 rats) in the RAGE-Ctrl group and 80% (16/20 rats) in the RAGE-Tx group. At 7 days following crush injury, the survival rate was 10% (2/20 rats) in the Ctrl group and 50% (10/20 rats) in the RAGE-Tx group. Seven-day survival was significantly improved in the RAGE-Tx group compared with the RAGE-Ctrl group (*P* < 0.01).

#### Serum levels of related biological parameters after crush injury

**Figure 3:**
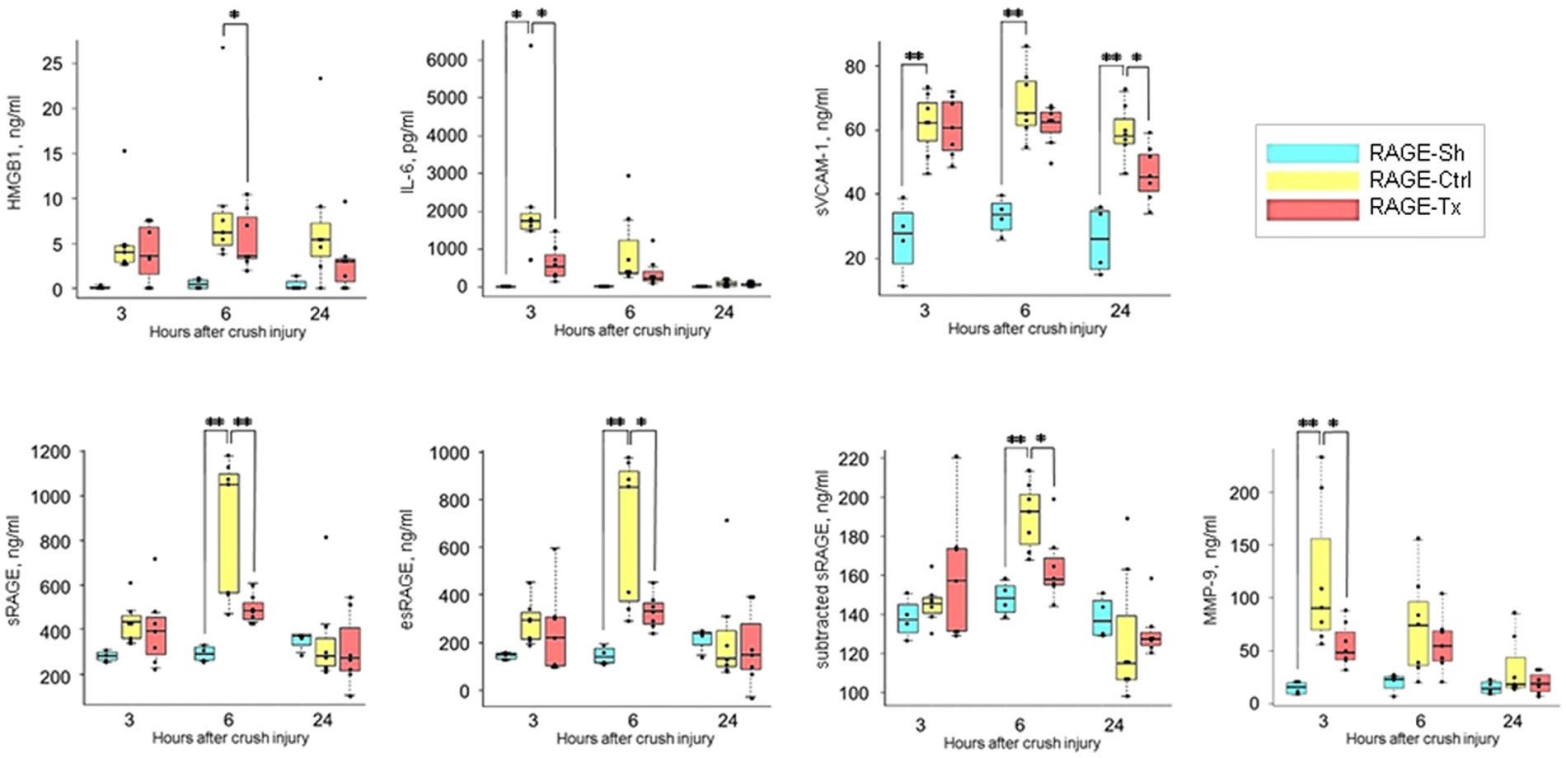
Changes in serum levels of related biological parameters obtained by ELISA after crush injury at 3, 6 and 24 h in the RAGE-Sh, RAGE-Ctrl and RAGE-Tx groups. (**A**) High-mobility group box 1 (HMGB1), (**B**) interleukin 6 (IL-6), (**C**) soluble vascular adhesion molecule 1 (sVCAM-1), (**D**) soluble RAGE (sRAGE), (**E**) endogenous secreted form of RAGE (esRAGE), (**F**) subtracted sRAGE and (**G**) matrix metallopeptidase 9 (MMP-9). RAGE-Sh group (n = 4), RAGE-Ctrl group (n = 7), RAGE-Tx group (n = 7). All data are expressed as median and interquartile range. All individual data points are shown for each parameter. ^*^ *P* < 0.05, ^**^ *P* < 0.01.

We assessed the serum HMGB-1 levels, which is one of the DAMPs. Significant increases of the serum HMGB-1 levels in the RAGE-Ctrl group were observed after crush injury at 6 h compared with those in the RAGE-Sh group (*P* < 0.05) (Fig. 3B). The level of IL-6 was measured to study the changes in serum levels of systemic inflammatory markers. Significant increases of the serum IL-6 levels in the RAGE-Ctrl group were observed after crush injury at 3 h compared with those in the RAGE-Sh group (*P* < 0.05), whereas the increases were significantly inhibited at 3 h after crush injury in the RAGE-Tx group (*P* < 0.05) (Fig. 3C). Endothelial activation promotes leukocyte endothelial adhesion via endothelial adhesion molecules such as VCAM-1. sVCAM-1 is released in response to an endothelial injury and used as an endothelial injury marker. Serum levels of sVCAM-1 in the RAGE-Ctrl group increased significantly after crush injury at all time points in comparison with those in the RAGE-Sh group. Serum sVCAM-1 levels at 24 h were significantly inhibited in the RAGE-Tx group compared with those in the RAGE-Ctrl group (*P* < 0.05) (Fig. 3D). Accumulating evidence suggests that the value of circulating sRAGE reflects the degree of total RAGE expression in the body. Therefore, we also evaluated the changes in serum levels of sRAGE. Significant increases of the serum sRAGE levels in the RAGE-Ctrl group were observed after crush injury at 6 h in comparison with those in the RAGE-Sh group (*P* < 0.01). Significant inhibition of the increases was observed at 6 h after crush injury by administration of the anti-RAGE antibody (*P* < 0.01) (Fig. 3E). sRAGE is composed of the esRAGE and cRAGE isoforms. An estimated value of cRAGE was calculated from the actual measured values of sRAGE and esRAGE (i.e. sRAGE – esRAGE) and reported as subtracted sRAGE. Significant increases of serum levels of esRAGE and subtracted sRAGE in the RAGE-Ctrl group were observed after crush injury at 6 h. However, significant inhibition of the increases was observed at 6 h after crush injury in the RAGE-Tx group (*P* < 0.05) (Fig. 3F,G). To estimate the changes in serum levels of proteolytic enzymes, we estimated the serum levels of MMP-9, which produces cleavage of the membrane-bound RAGE. The increase in MMP-9 in the Tx group was significantly inhibited at 3 h after crush injury in comparison with that in the RAGE-Ctrl group (*P* < 0.05) (Fig. 3H).

#### Haematoxylin-eosin staining to evaluate remote organ damage after crush injury

**Figure 4:**
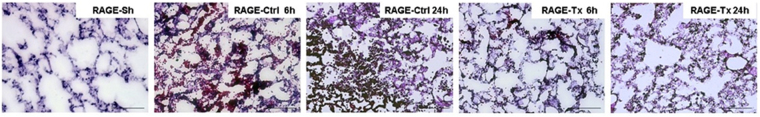
Haematoxylin-eosin staining of the lung after crush injury in the RAGE-Sh, RAGE-Ctrl and RAGE-Tx groups. Alveolar oedema and haemorrhage, inflammatory cell infiltration and disruption of the pulmonary architecture were present in the lungs of rats in the RAGE-Ctrl group after crush injury. These findings were reduced in the RAGE-Tx group compared with the RAGE-Ctrl group. Bars represent 100 µm. Original magnification ×200.

The lung tissues in the RAGE-Ctrl at 6 h after crush injury showed alveolar oedema and haemorrhage, inflammatory cell infiltration and disruption of the pulmonary architecture. This damage was also more obvious at 24 h after crush injury. These findings were reduced in the RAGE-Tx group, suggesting that administration of anti-RAGE antibody has an inhibitory effect on acute lung damage following crush injury (Fig. 4). The kidney tissues of the rats in the RAGE-Ctrl and Tx groups at 6 h after crush injury showed slight interstitial oedema and haemorrhage. However, there were no discernible changes between the two groups (data not shown), nor were there any morphological differences in the brains and livers of the three groups (data not shown).

#### Fluorescent immunostaining to detect RAGE expression

**Figure 5:**
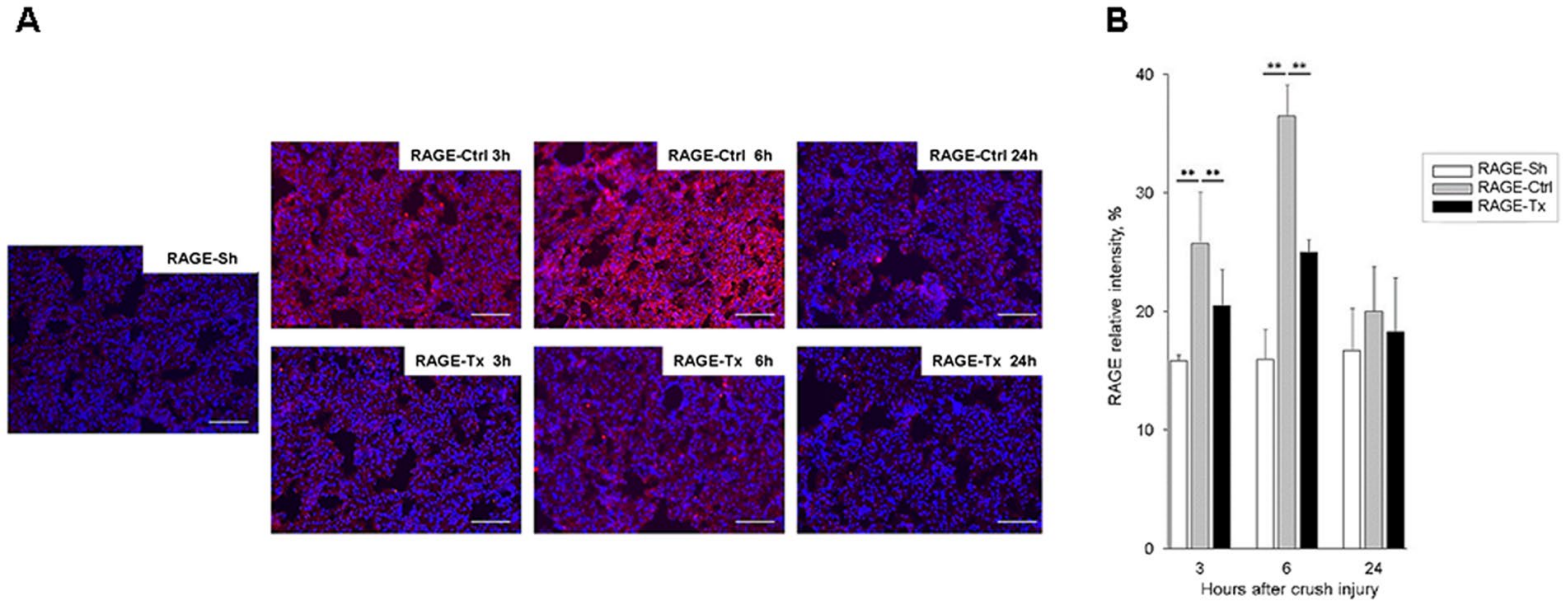
Immunofluorescent RAGE expression in the lung alveoli following crush injury. (**A**) The red signal indicates the expression of RAGE, and the blue signal indicates the location of the nucleus. In the lung, markedly upregulated RAGE in the RAGE-Ctrl group was suppressed by the administration of anti-RAGE antibody in the RAGE-Tx group at both times compared with that in the RAGE-Ctrl group. Bars represent 100 µm. Original magnification ×200. (**B**) Results of statistical analysis of the relative intensity of RAGE. Relative intensity was calculated by averaging fluorescent immunostaining in 10 randomly selected slide fields in the lung. All data are expressed as mean ± SD. RAGE-Sh group (n = 3), RAGE-Ctrl group (n = 4), RAGE-Tx group (n = 4). **P < 0.01.

**Figure 6:**
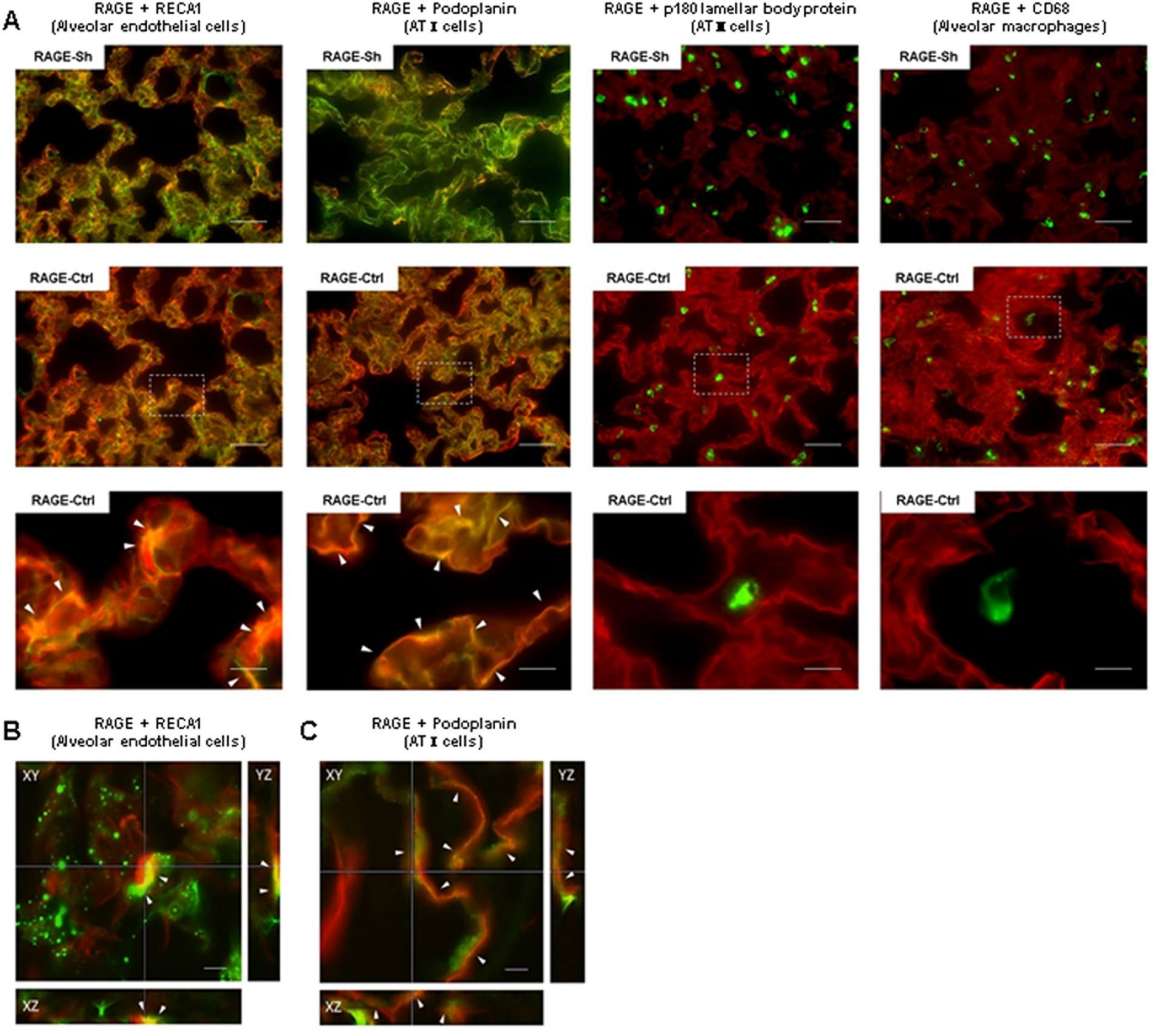
Immunohistochemical analysis to identify the localisation of RAGE expression in the lung alveoli. The red signal indicates the expression of RAGE. The green signal indicates the expression of RECA1-positive cells (alveolar endothelial cells), podoplanin-positive cells (ATI cells), p180 lamellar body protein-positive cells (ATII cells) and CD68-positive cells (alveolar macrophages), respectively. (**A**) The white dashed square shows the region from which the higher magnification image was taken. The white arrowheads in the respective panels indicate enhanced expression of RAGE in response to crush injury in endothelial cells and ATI cells. Expressions of RAGE were not enhanced on the ATII cells and alveolar macrophages. Bars represent 50 µm in the top and middle panels (original magnification ×400) and 10 µm in the bottom panels (original magnification ×2000). (**B**,**C**) The images in the lateral (X-Y) and axial (Z) direction are shown. The white arrowheads in the respective panels indicate the co-localization between RAGE and alveolar endothelial cells (**B**) and ATI cells at the plasma membrane (**C**). XY, YZ and XZ indicate the X-Y, Y-Z and X-Z images respectively. Bars represent 10 µm in the panels (original magnification ×1000).

The RAGE expression was enhanced in the rat lungs of the RAGE-Ctrl group: it was strongly upregulated at 6 h after crush injury and then reduced at 24 h. An inhibitory effect on RAGE expression induced by the administration of anti-RAGE antibody was observed in the RAGE-Tx group particularly at 6 h after crush injury compared with that in the RAGE-Ctrl group (Fig. 5A). The relative intensities of RAGE in the lungs in the RAGE-Ctrl group increased significantly after crush injury at 3 and 6 h compared with those in the RAGE-Sh group. Significant suppression of the increases was observed at 3 and 6 h after crush injury with the administration of anti-RAGE antibody in the RAGE-Tx group (*P* < 0.01) (Fig. 5B). To determine the cell population that is responsible for RAGE expression in the lung alveoli, immunohistochemical analysis was performed in the RAGE-Sh and RAGE Ctrl groups with anti-RECA-1 antibody, anti-podoplanin antibody (alveolar type I epithelial cell [ATI cell] marker), anti-p180 lamellar body protein antibody (alveolar type II epithelial cell [ATII cell] marker) and anti-CD68 antibody (alveolar macrophage marker). In the normal condition, basic RAGE expression was detected in alveolar endothelial cells and ATI cells, and the expression was enhanced in these cell types in response to the crush injury. ATII cells and alveolar macrophages showed no explicit immunoreactivity of RAGE in either group (Fig. 6). In the kidney, the RAGE expression was slightly upregulated at 6 h after crush injury, whereas in the RAGE Tx group, the upregulated RAGE expression was suppressed by the administration of anti-RAGE antibody (data not shown). Differences in RAGE expression were not recognised in the brains and livers of the three groups (data not shown).

### Experiment 2

#### Serum IL6 levels after crush injury

Serum levels of IL-6 in the TLR4-Ctrl group increased significantly after crush injury at 6 h compared with those in the TLR4-Tx group (*P* < 0.05) (Supplemental Fig. 1).

#### Haematoxylin-eosin staining to evaluate remote organ damages after crush injury

The lung tissues in the TLR4-Ctrl group at 6 h after crush injury showed alveolar oedema and inflammatory cell partially present in the alveolar space as well as the interstitial space. These findings were attenuated in the TLR4-Tx group, indicating that the administration of TLR4 antagonist has an inhibitory effect on the acute inflammation and subsequent acute lung injury following crush injury (Supplemental Fig. 2).

## Discussion

This is the first report, to our knowledge, to evaluate the association between RAGE signalling and pathologic conditions in a rat model of crush injury including systemic inflammation to assess the potential therapeutic effects of therapy with anti-RAGE antibody. The goal of this study was to develop new therapeutic agents for treating crush injury at the scene and to improve the survival rate of the injured.

Binding of ligands such as HMGB-1 to RAGE on cell membranes stimulates RAGE signalling via nuclear factor kappa B (NF-κB)^16,30^. HMGB1-RAGE signalling activates NF-κB through several pathways such as the ERK1/2 MAP kinase pathway in acute inflammatory diseases^18,31,32^. The activated RAGE signalling via NF-κB following ligand recognition of the RAGE receptor induces transcription of the RAGE gene per se leading to the upregulation of RAGE expression in a positive feedback manner^33^. The serum HMGB-1 levels increased immediately at 3 h after crush injury in the RAGE-Ctrl group and were significantly different between the Sh and RAGE-Ctrl groups when analysed by *t*-test (*P* = 0.049). Also, the relative intensity of RAGE in the lungs significantly increased at 3 h after crush injury. This suggests that the immediate extracellular release of HMGB-1 into the circulation from the damaged lower extremities just after the removal of the weights results in the binding of HMGB-1 to RAGE on cell membranes in the lung and promotes the positive feedback upregulation of RAGE expression in the mechanism described above.

In our study, the administration of anti-RAGE-antibody ameliorated the lung damage by suppressing the increase in RAGE expression in the lung alveoli. This suggests that RAGE expression is strongly associated with the lung damage. Next, we assessed the source of the RAGE expression at the cellular level in the lung. We found that RAGE expression was upregulated in both alveolar endothelial cells and ATI cells but not in ATII cells and alveolar macrophages after crush injury. It might be reasonable to consider that the first cell population that recognises RAGE ligands in the circulation generated by crush injury is the endothelium, and the subsequent endothelial activation provides an initial inflammatory milieu in the lung. In turn, this and the influx of RAGE ligands through the disrupted endothelial barrier would trigger dysfunction of the alveolar epithelial barrier leading to explosive progression of acute lung damage by augmenting RAGE expression in ATI cells because incremental expression of RAGE in ATI cells is reported to be an indicator of cell injury^19,34,35^.

Our results showed only a slight increase in the real amount of esRAGE (Fig. 3F). This suggests that the increase in sRAGE was mainly composed of cRAGE, which could be defined as subtracted sRAGE (sRAGE – esRAGE), and not of esRAGE. Also, the increase in the level of subtracted sRAGE peaked later than the surge of MMP in the RAGE-Ctrl group. These results suggest that MMP9 cleaved the cell membrane-anchored RAGE, and subsequently, the cRAGE was released into the circulation. In addition, it has been reported that serum cRAGE levels are more strongly associated with inflammation in the acute phase of trauma and sepsis than are esRAGE levels^21^. Therefore, our finding suggests the usefulness of also measuring cRAGE as a biomarker to evaluate the inflammatory response in the acute phase after crush injury.

The administration of anti-RAGE antibody ameliorated the crush injury-induced lung damage and improved the survival rate of the rats with crush injury. Activated RAGE signalling via NF-κB enhances the synthesis of certain cytokines such as IL-6 and endothelial adhesion molecules like VCAM-1 along with the upregulation of RAGE expression^36,37^. In our study, the relative intensity of RAGE in the lungs and the serum levels of IL-6 and sVCAM-1 increased significantly at 3 h after crush injury (*P* < 0.05 and *P* < 0.01, respectively), and this reaction was hampered by anti-RAGE antibody treatment. Early intervention with the administration of anti-RAGE antibody might block this signalling pathway before substantial induction of the inflammatory response, thus interrupting a vicious cycle of positive feedback expression of RAGE.

The HMGB1 that binds to RAGE and to TLR2 and TLR4 ends up activating NFkB^38^. In this study, we evaluated the effectiveness of the TLR4 inhibitor to validate the influence of the TLR4 signal. The administration of the TLR4 antagonist ameliorated the synthesis of IL-6 levels and the crush injury-induced lung damage, suggesting that the TLR4 signal plays an important role along with the RAGE signal. This is supported by evidence showing that TLR-4 knock-out mice or RAGE knock-out mice displayed lower levels of TNF-α and IL-6 compared to the wild-type mice in HMGB-1-induced inflammation^39^. One possible reason is that the blockade of each of the signals reduces the induction of HMGB1, thus attenuating subsequent signal activation. Another possible reason is that RAGE and TLR4 signals mutually interact with each other. In fact, several articles have reported possible crosstalk between the RAGE and TLR4 signals through intracellular adaptor proteins such as TIRAP and MyD88^17,40^. In addition, these findings could support the similar effectiveness of HMGB-1^10^ and RAGE blockades in the present rat model of crush injury. However, RAGE binds to a variety of ligands other the HMGB-1, and these ligands might shed light on the difference in the mechanism of action between HMGB-1 and RAGE blockades. Further study addressing the crosstalk between the RAGE, TLR2 and TLR4 signals and investigation into the ligands of RAGE will be needed.

Recent technological evolution has enabled the emergence of lifesaving services with the technology to intervene in treating crush injury patients before rescuing them from the wreckage at the scene of the disaster or accident. We believe that the administration of anti-RAGE antibody before releasing compression might be a useful treatment that could be administered to patients with crush injury at the scene.

The data in the present rat model of crush injury are in general consistent with those of prior reports from our group^8,10,27^, indicating the stability of this model. However, there are two limitations in this study. First, we only evaluated the effect of the administration of anti-RAGE antibody immediately before the removal of the weights on the assumption that the antibody would be administered at the scene. For administration of the antibody after patient arrival at the hospital, further study that investigates the effects of delayed administration of the anti-RAGE antibody on crush injury is required. Second, the anti-RAGE antibody used for treatment, which binds to the RAGE V domain, might also bind to that of sRAGE, including cRAGE and esRAGE, thus affecting the sRAGE measurements.

## Conclusions

The expressions of RAGE in the lung and of sRAGE (mainly composed of cRAGE) were increased in a rat model of crush injury, suggesting that RAGE signalling plays a crucial role in the induction of systemic inflammation and acute lung damage following crush injury. Intravenous administration of anti-RAGE antibody before releasing compression dampened systemic inflammation and improved survival, suggesting that the therapeutic use of anti-RAGE antibody might have a beneficial effect on the prognosis of crush injury by preventing the development of MOF.

## Electronic supplementary material

Supplemental information
