# Supplementary material for: Therapeutic Effectiveness of Anti-RAGE Antibody Administration in a Rat Model of Crush Injury
Source: Sci Rep. 2017 Sep 25;7:12255. doi: 10.1038/s41598-017-12065-4 (PMC5613014; doi:10.1038/s41598-017-12065-4)
Supplement: Supplementary file 1 — Supplemental information [file 41598_2017_12065_MOESM1_ESM.pdf]

Research Article

Therapeutic Effectiveness of Anti-RAGE Antibody Administration in a Rat Model of Crush Injury

Hisatake Matsumoto[1*], Naoya Matsumoto[1], Junya Shimazaki[1], Junichiro Nakagawa[1], Yukio Imamura[2], Kazuma Yamakawa[1], Tomoki Yamada[1], Mitsunori Ikeda[1], Hiroko Hiraike[1], Hiroshi Ogura[1] and Takeshi Shimazu[1]

[1]Department of Traumatology and Acute Critical Medicine, Osaka University Graduate School of Medicine, 2-15 Yamadaoka Suita, Osaka 565-0871, Japan

[2]Laboratory of Nano-Bio Probes, RIKEN Quantitative Biology Center, 6-2-3 Furuedai, Suita, Osaka 565-0874, Japan

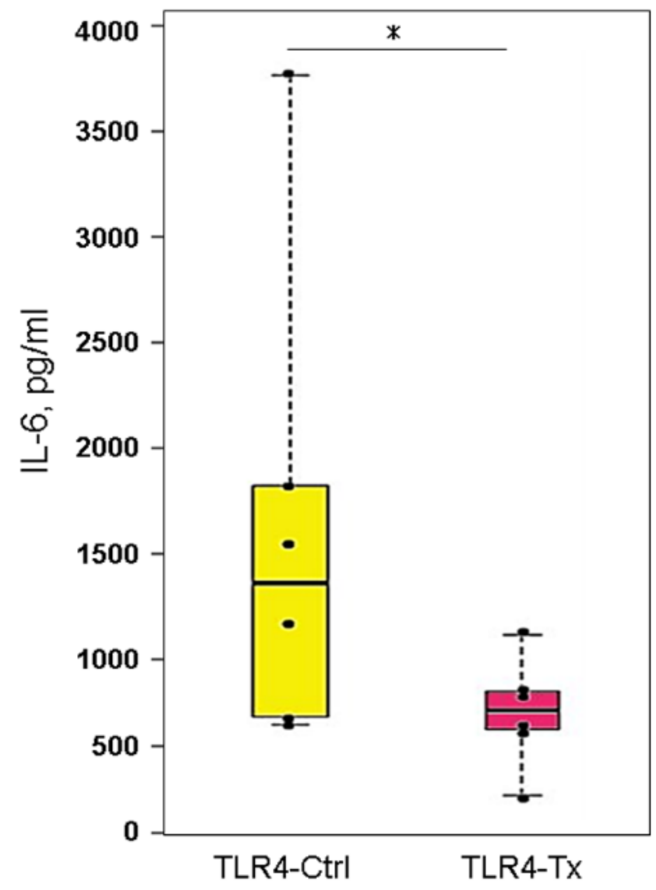

Supplemental Figure 1. Serum levels of interleukin-6 (IL-6) obtained by ELISA after 6 h of crush injury in the TLR4-Ctrl and TLR4-Tx groups. TLR4-Ctrl group (n = 6), TLR4-Tx group (n = 6). All data are expressed as the median and interquartile range. All individual data points are shown for each parameter. *$P < 0.05$.

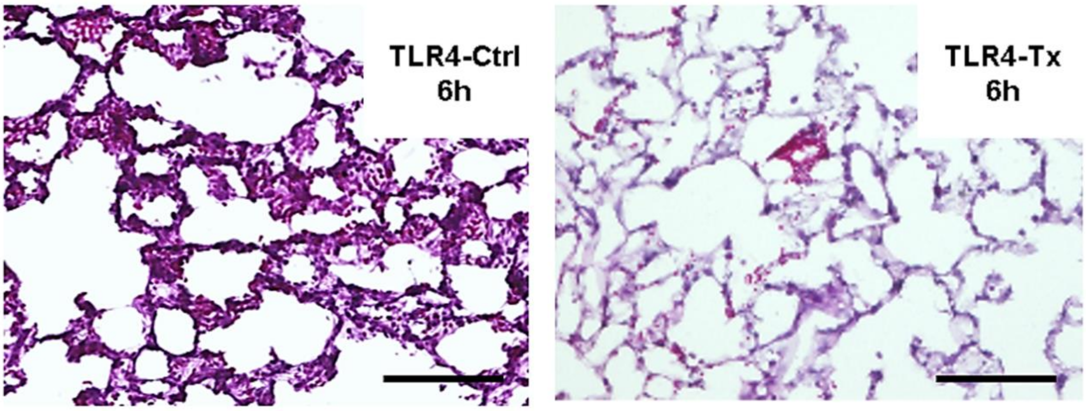

Supplemental Figure 2. Haematoxylin-eosin staining of the lung after crush injury in the TLR4-Ctrl and TLR4-Tx groups. Alveolar oedema and inflammatory cells partially in the alveolar space and in the interstitial space were seen in the lungs of the TLR4-Ctrl group after crush injury. These findings were reduced in the TLR4-Tx group compared with the TLR4-Ctrl group. Bars represent 100 μm. Original magnification ×200.
